# Ultra-High-Frequency Ultrasound in the Evaluation of Paediatric Pilomatricoma Based on the Histopathologic Classification

**DOI:** 10.3389/fmed.2021.673861

**Published:** 2021-04-26

**Authors:** Li Li, Jiaosheng Xu, Siwei Wang, Jun Yang

**Affiliations:** ^1^Department of Dermatology, Plastic Surgery Hospital, Chinese Academy of Medical Sciences and Peking Union Medical College, Beijing, China; ^2^Department of Dermatology, Beijing Children's Hospital, National Center for Children's Health, Capital Medical University, Beijing, China; ^3^Department of Radiology, Beijing Children's Hospital, National Center for Children's Health, Capital Medical University, Beijing, China; ^4^Institute of Biomedical Engineering, Chinese Academy of Medical Sciences, Tianjin, China

**Keywords:** pilomatricoma, ultrasound, ultra-high-frequency, diagnosis, paediatric, histopathologic

## Abstract

**Background:** Pilomatricoma (PM) is one of the most common benign tumours in children. However, the inaccuracy of preoperative diagnosis and evaluation is high. Non-invasive examinations, including dermoscopy and ultrasound are helpful for diagnosing and evaluating PM. To date, ultra-high-frequency ultrasonographic features of PM have been rarely studied.

**Objective:** We aimed to investigate the ultra-high frequency ultrasonographic features of PM in a large paediatric cohort and to determine the associations of these features with the clinical features of different histological subtypes of PM.

**Methods:** This was a retrospective study. Patients who had both preoperative ultra-high-frequency ultrasonographic evaluation and pathological diagnosis of PM were enrolled. A series of infantile haemangiomas and cutaneous cysts during the same period were included as controls. Histological findings, including the stage, calcifying type, and ultrasonographic features of each lesion, were described.

**Results:** A total of 133 patients with PM were included, and 147 PM lesions were analysed. The male-to-female ratio was 1:1.58, and the median age of onset was 91 (range: 10–188) months. On ultra-high-frequency ultrasonography, PM presented as heterogeneous (144/147, 98.0%), well-demarcated (143/147, 97.3%), and hypoechoic (126/147, 85.7%) tumours located between the deep dermis and subcutaneous tissue (139/147, 94.6%). The most common features were internal echogenic foci (135/147, 91.8%), hypoechoic rim (133/147, 90.5%), and posterior acoustic shadowing (94/147, 63.9%). Fourteen (9.5%) lesions were histologically categorized in the early stage, 58 (39.5%) in the fully developed stage, 65 (44.2%) in the early regressive stage and 10 (6.8%) in the late regressive stage. Three calcifying types, including scattered dots, clumps and arcs, were observed on histologic examination, which corresponded well with grey-scale imaging on ultra-high-frequency ultrasonography. Each calcifying type was significantly different in various histological stages (*P* = 0.001), among which scattered dots were mainly present in the early and fully developed stage and arc-shaped calcifying were present in the regressive stages. Calcification was observed in skin cysts, while there was more frequent posterior enhancement, less frequent posterior shadowing, and hypoechoic rim than PM. Haemangioma also presented as a hypoechoic tumour on grey-scale imaging. However, haemangioma was homogeneous and rarely calcifying.

**Conclusions:** PM is a heterogeneous, well-demarcated, hypoechoic tumour located between the deep dermis and the subcutis on ultra-high-frequency ultrasonography. The most common features are internal echogenic foci (calcifying) and hypoechoic rim. Calcifying types can help in the staging of PM. Ultra-high-frequency ultrasound is a useful tool for the diagnosis and evaluation of PM.

## Introduction

Pilomatricoma (PM) is a benign, deep dermal or subcutaneous tumour that is derived from the hair cortex cell (1). It is also known as calcifying epithelioma of Malherbe (2). PM has a bimodal peak, first presenting in the first two decades and second in the sixth decade. It has a female predominance. Lesions mainly occur on the head, neck, and extremities, which range from 0.5 to 3 cm in size. On palpation, PM is characteristically hard and nodular or multinodular. The overlying skin can be bluish or reddish, which can mimic infantile haemangioma (3).

The typical histopathology of PM shows sheets of basaloid cells and masses of eosinophilic cornified material containing ghost cells. Calcification is mostly observed in the ghost cell regions. Based on morphological features, PM is classified into four stages (4). The first is the early stage and is typified by small cystic structures lined by squamoid and basaloid epithelium, and shadow cell formation. The second is the fully developed stage characterized by large neoplasms lined by basaloid epithelium at their periphery; within, it comprises clusters of shadow cells. The third is the early regressive stage with no apparent epithelial lining but has basaloid cell foci at the periphery; within, it comprises shadow cells surrounded by granulation tissue. The fourth is the late regressive stage, demonstrating no obvious parenchymal cells but irregularly shaped, partially confluent masses of faulty hair material, and calcification or ossification, with little or no inflammatory infiltrate.

The clinical diagnosis rate of PM is usually very low. Prior reviews have shown that preoperative diagnostic accuracy for PMs ranges from 21 to 32% (1, 5). The most common differential diagnoses are haemangiomas and several types of skin cysts (3, 6). Non-invasive techniques such as dermoscopy and ultrasonography have been increasingly used to aid in the diagnosis of skin tumours (7, 8). They are popular in paediatric patients because they are simple, quick, and non-invasive. Hughes et al. reported on the sonographic diagnosis of PM in childhood with a diagnostic accuracy of 80% (9). On ultrasound, PM is a heterogeneous tumour with inner echogenic foci and a hypoechoic rim or a completely echogenic mass with strong posterior acoustic shadowing in the subcutis (10). The reliable diagnostic features for PM are hypoechogenicity, heterogenicity, internal echogenic foci, hypoechoic rim, and posterior shadowing (11).

However, another study has demonstrated PM as a well-defined hyperechoic or calcific nodule in subcutaneous fat (12), probably because of the different frequencies of the transducer. Therefore, we used ultra-high-frequency ultrasound to explore the ultrasonographic features and determine the histopathological correlations of PM.

## Materials and Methods

This was a retrospective study. From April 2016 to December 2020, paediatric patients with histopathologically proven PM after surgery at the Plastic Surgery Hospital, Chinese Academy of Medical Sciences and Peking Union Medical College and Beijing Children's Hospital, Capital Medical University, were included in this study with Institutional Review Board approval. Written informed consent for the procedure was obtained from all the patients' parents. A series of 91 infantile haemangiomas and 28 cutaneous cysts (including dermal and epidermal cysts) during the same period were included as controls.

### Ultra-High-Frequency Ultrasonography

Ultrasonographic examination was performed using an ultrasound skin scanner (MD-300S, MEDA, Co., Ltd., Tianjin, China) equipped with 50 MHz linear array transducers. The grey-scale images were captured by the bioscope (MD-300SII, MEDA, Co., Ltd., Tianjin, China) attached to the ultrasonograph. Each image was reviewed by an experienced radiologist and dermatologist. The analysis of ultrasonographic features included tumour location, margin, shape, size, echo texture, and echogenicity. The tumour echo texture was recorded as homogeneous or heterogeneous, and echogenicity as hyperechoic if the tumour echogenicity was higher than that of dermal connective tissues. The following ultrasonographic features were observed in the study: inner echogenic foci, posterior shadowing, hypoechoic rim, and peritumoral hyperechogenicity. Inner echogenic foci were defined as calcification regardless of posterior shadowing. The calcification mode was described as an arc, scattered dots, or clumps.

### Histopathology

After ultrasonographic examination, the tumours were arranged for complete resection and routine histological examination. Each lesion was examined by the experienced pathologist and dermatologist. Based on the morphology of tumour and infiltrating cells, the histopathological stages were categorized into four stages as in a previous report (4): early, fully developed, early regressive, or late regressive stage. The calcification shape at low magnification was described as scattered dots, an arc, or clumps.

### Statistical Analyses

Descriptive statistics for categorical variables are presented as numbers. The differences between two independent categorical variables were evaluated using the chi-square test (Fisher exact test when necessary) and Kruskal-Wallis rank sum tests were performed for grouped data. SPSS Windows version 26.0 (SPSS Inc., Chicago, IL, USA) was used for the statistical analyses and *P* < 0.05 was considered statistically significant.

## Results

All patients underwent preoperative ultra-high-frequency ultrasound examination and were histologically diagnosed with PM post-operatively.

### Clinical and Histopathological Findings of PM

**Figure 1:**
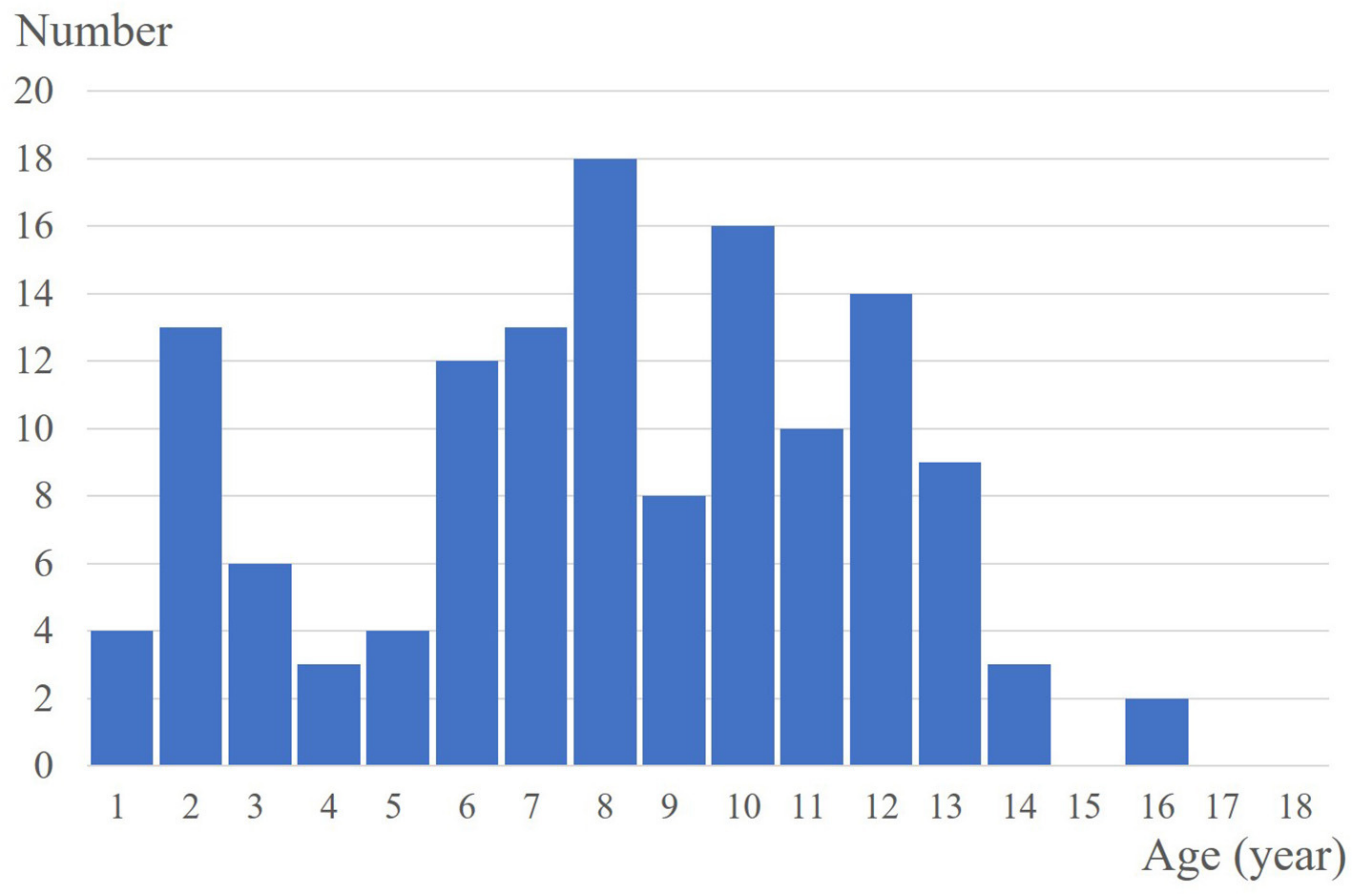
Population distribution with age in pediatric PM.

A total of 133 patients with PM were included, and 147 PM lesions were analysed. The male-to-female ratio was 1:1.58. The median age of onset was 91 (range: 10–188) months, and two peaks were observed (ages 2 and 8) (Figure 1). The meantime from onset to diagnosis was 14.8 months (range: 0.5–132 months). One hundred and twenty-two patients had a solitary lesion, eight patients had two lesions, and three patients had three lesions (113 in the head and neck, 11 in the trunk and 23 in the extremities). The mean length of the largest tumour diameter was 0.8 (range: 0.3–7) cm. Fourteen (9.5%) lesions were histologically categorized in the early stage (**Figure 3A**), 58 (39.5%) in the fully developed stage (**Figure 4A**), 65 (44.2%) in the early regressive stage (**Figure 5A**), and 10 (6.8%) in the late regressive stage (**Figure 6A** and Table 1). Three calcifying types, including scattered dots, clumps and arcs, were observed on low power histologically. On high power, bone formation was noted in the arc-shaped calcification but not in the other two types.

**Table 1:** Ultra-high-frequency ultrasonographic features in various histological stages.

**Ultrasound features**	**Histological staging**				P^*^
	**Early (*n* = 14)**	**Fully developed (*n* = 58)**	**Early regressive (*n* = 65)**	**Late regressive (*n* = 10)**	
**Echogenicity**					
Hyperechoic	2	8	11	0	
Hypoechoic	12	50	54	10	
**Echotexture**					
Homogeneous	2	1	0	0	
Heterogeneous	12	57	65	10	
**Margin**					0.001
Well	13	57	63	10	
Ill	1	1	2	0	
**Calcification**	8	53	60	10	
Scattered dots	5	34	20	0	
Clumps	3	17	28	4	
An arc	0	2	12	6	
**Hypoechoic rim**	13	57	63	10	
**Posterior shadowing**	1	30	55	8	
**Posterior enhancement**	3	6	0	0	
**Peritumoral hyperechogenicity**	2	23	18	2	

* *Kruskal-Wallis rank-sum test*.

### Ultrasonographic Features of PM and Its Clinical and Histological Correlations

**Figure 2:**
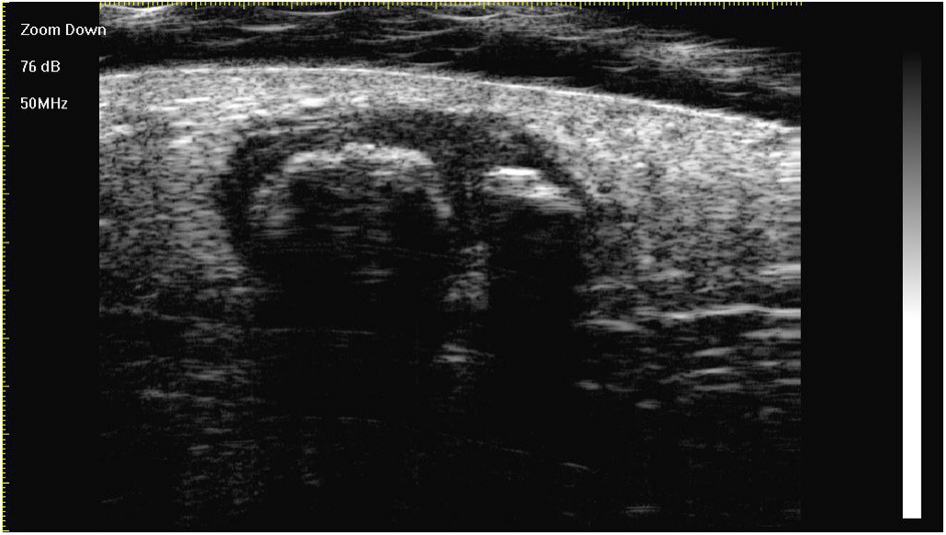
The lesion is a heterogeneous, well-demarcated, hypoechoic tumour located between the deep dermis and the subcutis with typical ultra-high-frequency ultrasonographic features, including internal echogenic foci, hypoechoic rim, and posterior acoustic shadowing.

**Figure 3:**
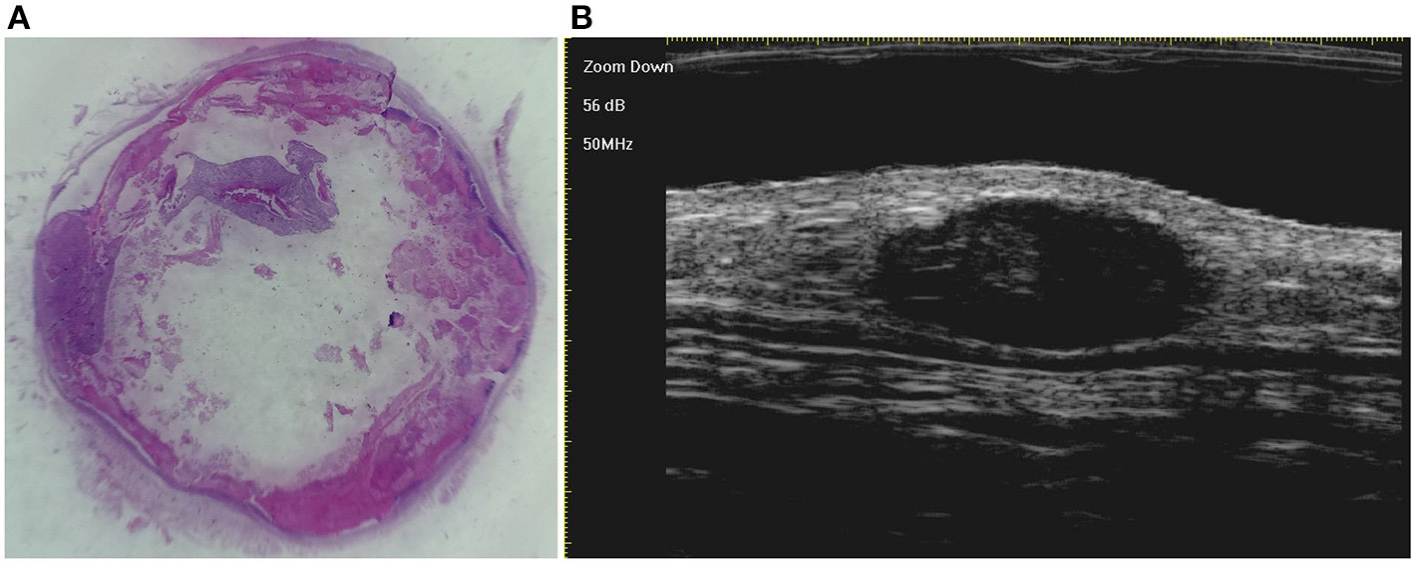
The histological presentation [**(A)** HE, original magnification × 10] and ultra-high frequency ultrasound **(B)** of PM in the early stage, indicating no obvious calcification and cystic formation.

**Figure 4:**
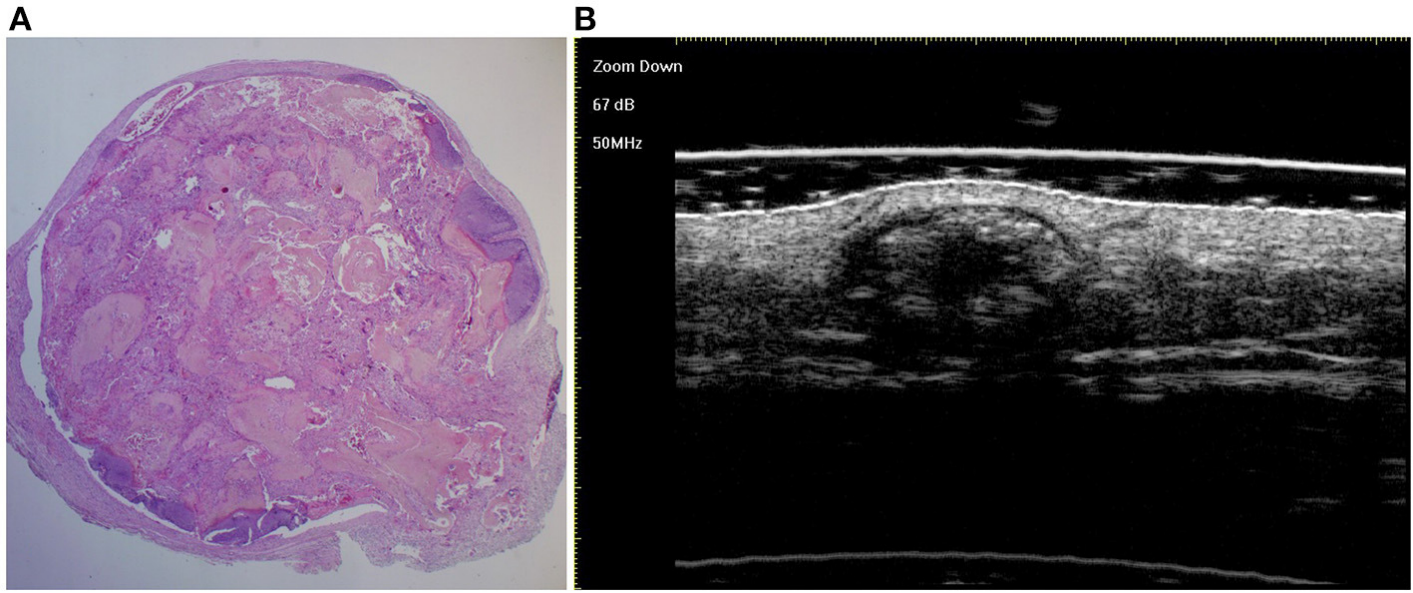
The histological presentation [**(A)** HE, original magnification × 25] and ultra-high frequency ultrasound **(B)** of PM in the fully developed stage, indicating scattered dot calcification.

**Figure 5:**
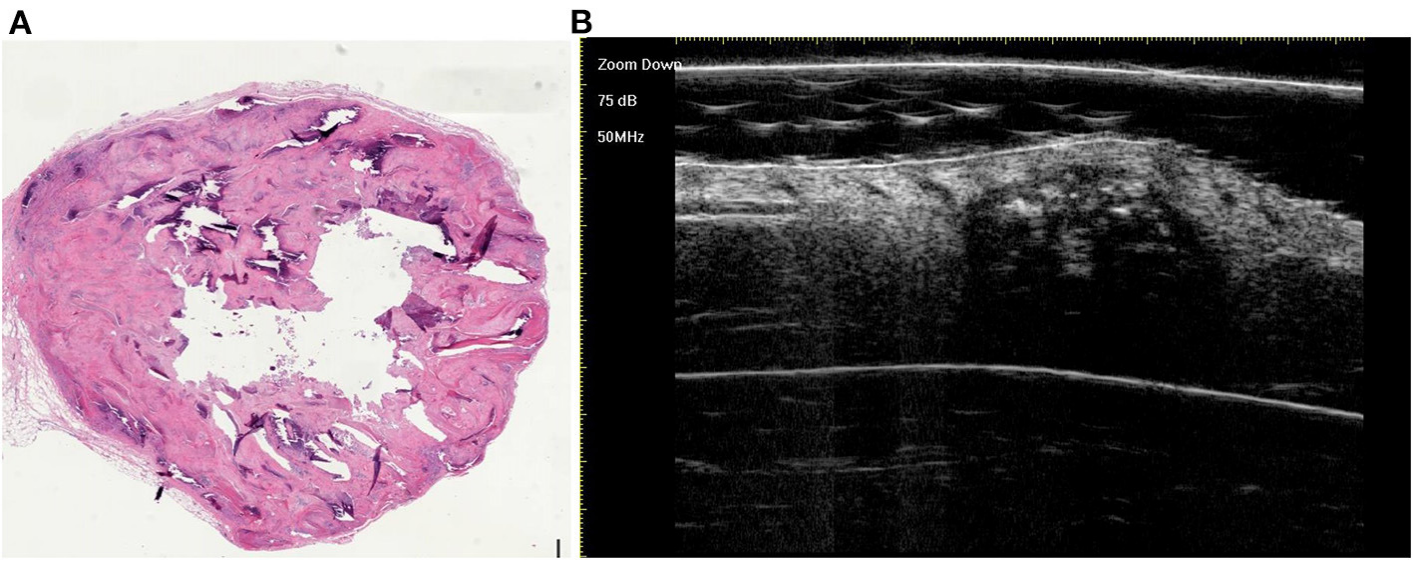
The histological presentation [**(A)** HE, original magnification × 10] and ultra-high frequency ultrasound **(B)** of PM in the early regressive stage, indicating clump calcification.

**Figure 6:**
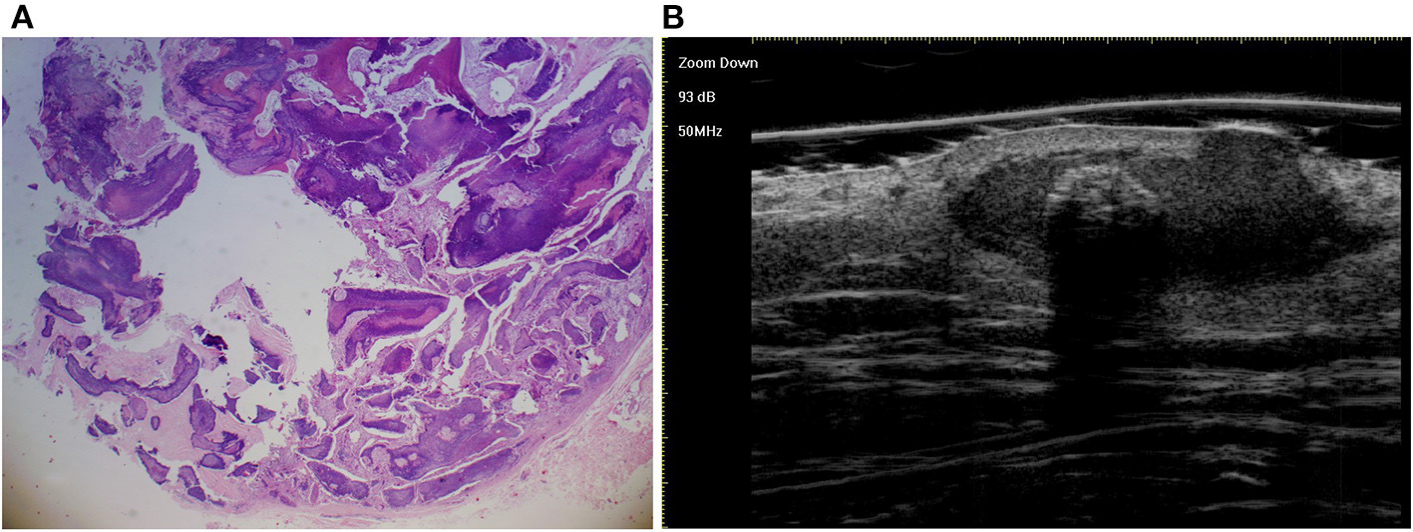
The histological presentation [**(A)** HE, original magnification × 25] and ultra-high frequency ultrasound **(B)** of PM in the early regressive stage, indicating arc calcification.

**Figure 7:**
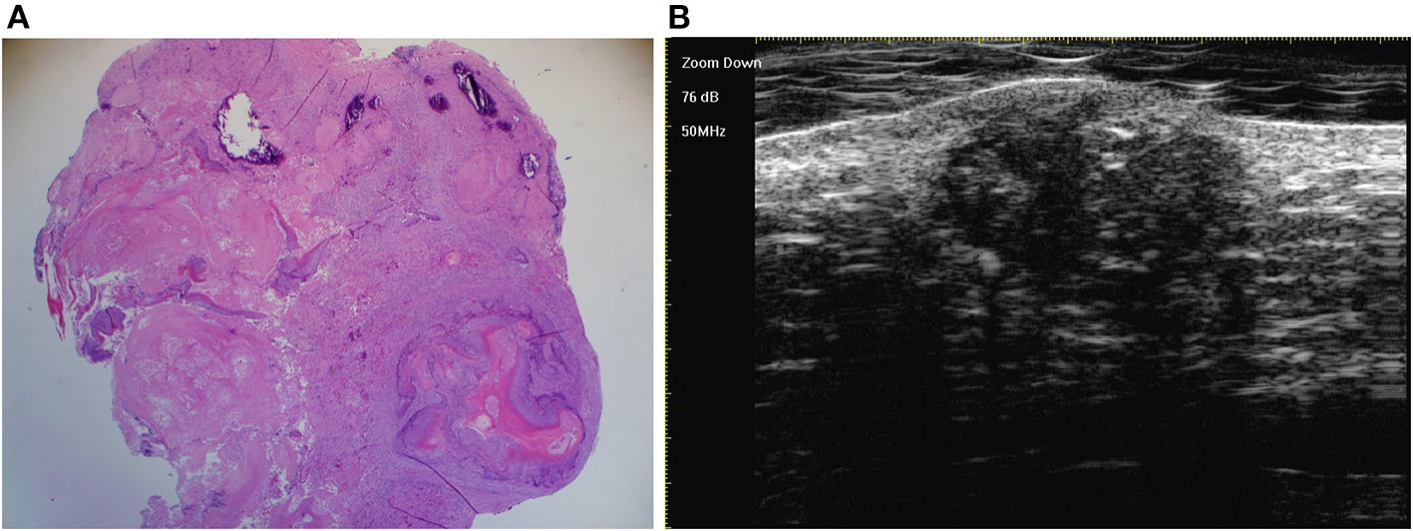
The last three stages overlap in one lesion, which is detected by histology [**(A)** original magnification × 25] and ultra-high frequency ultrasound **(B)**.

On ultra-high frequency ultrasonography, PM manifested as heterogeneous (144/147, 98.0%), well-demarcated (143/147, 97.3%), and hypoechoic (126/147, 85.7%) tumours located between the deep dermis and subcutaneous tissue (139/147, 94.6%) (Table 2). The lesions were mostly arranged in a regular shape (oval or round), while a few were irregular in shape. The most common features were internal echogenic foci (135/147, 91.8%), hypoechoic rim (133/147, 90.5%), posterior shadowing (94/147, 63.9%), and peritumoral hyperechogenicity (45/147, 30.6%) (Figure 2). Three calcifying types, including scattered dots, clumps, and arcs, were observed on ultra-high frequency ultrasonography corresponding well with the histological manifestations (Figures 3B, 4B, 5B, 6B). Each calcifying type was significantly different in the various histological stages (*P* < 0.05), among which scattered dots were mainly presented in the early and developed stages, while arc-shaped calcifications were present in the regressive stages. The different stages were observed in one lesion (Figure 7). Posterior enhancement was observed only in the early (3/14) and fully developed (6/58) stages, when cystic architecture formation was detected histologically (Table 2).

**Table 2:** Comparison of PM with hemangioma and skin cysts on ultrasound.

**Ultrasonographic features**	**PM**	**Hemangioma**	P^*^	**PM**	**Cysts**	P^*^
**Shape**			0.001			NS
Round/oval	143	10		143	26	
Irregular	4	81		4	2	
**Location**			0.001			0.001
Dermis	8	79		8	19	
Dermis to subcutaneous layer	139	12		139	9	
**Largest diameter (mean, mm)**	0.8 (0.3–7)	0.7 (0.6–8)	NS	0.8 (0.3–7)	0.6 (0.5–1.2)	NS
**Echogenicity**			0.001			NS
Hyperechoic	21	1		21	11	
Hypoechoic	122	90		122	17	
**Echotexture**			0.001			0.001
Homogeneous	3	88		3	19	
Heterogeneous	140	3		140	9	
**Margin**			0.01			0.001
Well	143	81		143	20	
Ill	4	10		4	6	
**Internal echogenic foci**		0.001			0.001
Scattered dots	59	1		59	4	
Clumps	52	1		52	1	
An arc	20	0		20	0	
**Hypoechoic rim**	140	1	0.001	143	2	0.001
**Posterior shadowing**	94	0	0.001	94	3	0.001
**Posterior enhancement**	9	1	NS	9	24	0.001
**Peritumoral hyperechogenicity**	45	1	0.001	45	2	0.001

*NS, not significant; PM, pilomatricoma*.

* *Chi-square test*.

### Comparison With PM From Infantile Haemangioma and Skin Cysts

A total of 91 patients with infantile haemangioma and 28 patients with skin cysts were enrolled as controls. The male–to–female ratio was 1:2.6 and 1:0.87, respectively. The mean age of onset was 3 (range: 1–20) months and 41 (range: 3–204) months, respectively. Infantile haemangioma also presented as a well-defined (81/91, 89.0%), and hypoechoic tumour (90/91, 98.9%) on grey-scale imaging. However, infantile haemangiomas were irregular (81/91, 89.0%) in shape and homogenous (88/91, 96.7%), rarely with calcification (2/91, 2.2%), and mostly located in the dermis (79/91, 86.8%). In addition, hypoechoic rim, posterior shadowing, posterior enhancement, and peritumoral hyperechogenicity were rarely observed in infantile haemangiomas. Calcification was observed in skin cysts (5/28, 17.9%), while there was more frequent posterior enhancement (24/28, 85.7%), less frequent posterior shadowing (3/28, 10.7%), and less hypoechoic rim (2/28, 7.1%) than in PM (Table 2).

## Discussion

In our study of the paediatric population, PM had a predilection for girls and the lesions mostly presented as solitary nodules, mainly occurring in the head and neck area, as previously reported (12). However, two peaks of onset in paediatric population were observed in our study. PMs in children present a great challenge in clinical diagnosis and treatment, especially regarding the first peak onset age. Although PM is one of the most common skin tumours in children, its clinical diagnosis is very low (1, 6). The accurate diagnosis rate in accordance with post-operative histopathology is <35% (1). Ultrasonographic assessment can improve the diagnosis of PM (11, 13, 14).

The ultrasonographic features of PM have been described in several studies (9–13), and include calcification in the heterogeneous background of the hypoechoic area, posterior acoustic shadowing, hypoechoic rim, and peritumoral hyperechogenicity. The background of the hyperechoic areas was also described (12). These ultrasonographic features of PM were observed in the present study. According to our data, most of the cases were hypoechoic on ultra-high frequency ultrasonography. On histopathology, this hypoechoic area demonstrated infiltrates of basaloid, ghost cells, and reactive inflammation accordingly. The high ratio of hypoechogenicity was detected mainly due to the use of ultra-high-frequency transducers in our study, which improved the lesion tissue resolution (7).

The most common ultrasonographic features of PM are calcification and a hypoechoic rim compared with infantile haemangiomas and skin cysts. The ultra-high-frequency ultrasound is sensitive to display minimal calcification. Calcification on ultrasound mainly presented as scattered dots, clumps, and arcs, which is consistent with the previous reports (15). The morphology of the calcification is well-demonstrated on histopathology. Moreover, various histological stages notably displayed distinct calcifying types in our study. In the early and fully developed stages, scattered dots were the main type of calcification, whereas in the regressive stages, arc-shaped calcifications were the main type. Clumps can manifest in all the histological stages. Histopathologic examination reveals that the hypoechoic rim represents a connective tissue capsule surrounding the tumour. Unlike various types of skin cysts, there is no cystic wall in PM, which is lined with compact epithelial tissues. This annular connective loose connective tissue in PM was well-demonstrated characteristically on high frequency ultrasonography as a hypoechoic rim. The overall analysis of calcification and the hypoechoic rim can help in the diagnosis of PM. Moreover, the evolution of calcification reflects the PM process (4). The analysis of calcifying type on preoperative ultrasound may aid in the staging of PM.

Clinically, the overlying skin of some PMs may manifest as bruises or reddish hues. On ultrasound, infantile haemangioma also presents as a hypoechoic tumour, as in PM. Occasionally, PM may manifest as a confluent colour Doppler flow, mimicking haemangioma. However, infantile haemangioma often manifests in a homogeneous manner compared to PM and rarely displays calcification on grey-scale imaging. Calcification can be present in skin cysts on ultrasound. However, the calcifying type mainly present as scattered dots in the skin cysts. In addition, a hypoechoic rim is rarely present, while posterior acoustic enhancement is common in skin cysts.

The main method for PM management is surgical resection. However, surgery is usually not accepted for PM in young children because of the risk associated with surgery and systemic anaesthesia. Thus, ultra-high-frequency ultrasonography can be used to detect PM progression according to the calcifying type when a follow-up strategy is adopted. PM evolves into a regressive stage with age and can be resected with more safety at an older age. Moreover, preoperative evaluation of location, size, margin, and shape can be obtained by ultra-high-frequency ultrasonography.

In summary, PM is a heterogeneous, well-demarcated, hypoechoic tumour located between the deep dermis and subcutis on ultra-high-frequency ultrasonography. The most common features are calcification and a hypoechoic rim. Calcifying types can help in the staging of PM. Ultra-high-frequency ultrasound seems a useful tool for the diagnosis and evaluation of PM.

To our knowledge, this is the largest sample of PM used to explore ultra-high-frequency ultrasonographic characteristics based on histopathological stages. However, this study was limited by its retrospective nature and lack of vascularity analysis on colour Doppler ultrasonography. Further prospective studies with larger samples should be initiated to expatiate the results described herein.

## Data Availability Statement

The original contributions generated for the study are included in the article/supplementary material, further inquiries can be directed to the corresponding author/s.

## Ethics Statement

The studies involving human participants were reviewed and approved by Plastic Surgery Hospital, Chinese Academy of Medical Sciences and Beijing Children's Hospital, Capital Medical University. Written informed consent to participate in this study was provided by the participants' legal guardian/next of kin. Written informed consent was obtained from the individual(s), and minor(s)' legal guardian/next of kin, for the publication of any potentially identifiable images or data included in this article.

## Author Contributions

LL and JX conceptualized and designed the study, drafted the initial manuscript, and reviewed and revised the manuscript. JX conceptualized and designed the study, coordinated and supervised data collection, conducted the initial analyses, drafted the initial manuscript, and critically reviewed the manuscript for important intellectual content. SW and JY designed the data collection instruments, collected data, conducted the initial analyses, and reviewed and revised the manuscript. All authors approved the final manuscript as submitted and agree to be accountable for all aspects of the work.

## Conflict of Interest

The authors declare that the research was conducted in the absence of any commercial or financial relationships that could be construed as a potential conflict of interest.
